# Sebum levels are associated with the relationship between skin properties and microbiota in Japanese women

**DOI:** 10.1038/s41598-026-59379-w

**Published:** 2026-07-03

**Authors:** Yuki Ishii, Miyuki Kudo, Akemi Imaoka, Tatsuichiro Shima

**Affiliations:** Department of Cosmetic Research, Yakult Central Institute, 5-11, Izumi, Kunitachi-shi, Tokyo, 186-8650 Japan

**Keywords:** Skin properties, Skin microbiota, Sebum content, Skin aging, Menopausal status, Japanese women, Diseases, Health care, Medical research, Microbiology

## Abstract

The diversity of the skin microbiota in Japanese women increases with skin aging and is accompanied by decreased sebum content. However, the impact of changes in skin microbiota on skin aging remains unclear. We conducted a cross-sectional subgroup analysis based on age, menopausal status, and sebum content in a cohort of Japanese women aged 30–60 years to elucidate the differences in the relationship between skin properties and microbiota under various conditions. As a fundamental characteristic of this cohort, we confirmed that older participants had lower sebum content and a higher alpha-diversity index. Subgroup analysis based on menopausal status and sebum content revealed differences in the correlations between several skin properties and the conditional dependence structure estimated by Graphical Lasso in groups with varying sebum levels. Additionally, in groups with higher sebum levels, multiple bacterial genera were identified as significantly associated with pH, sebum content, and transepidermal water loss, whereas none were detected in groups with lower sebum levels. These findings suggest that sebum may be associated with differences in the relationship between skin properties and microbiota and may provide a basis for future studies on skincare approaches tailored to sebum levels and specific bacterial features or diversity.

**Supplementary Information:**

The online version contains supplementary material available at https://doi.org/10.1038/s41598-026-59379-w.

## Introduction

Skin conditions are closely related to aging, manifested as reduced skin elasticity and increased age spots and wrinkles^1^. These changes are influenced by the accumulation of external stimuli, including ultraviolet radiation, chemicals, temperature, and bacterial infection, as well as endogenous factors such as reduced cellular function and hormonal imbalance^2^. Menopausal symptoms in women significantly affect skin conditions, such as dryness, thinning, sagging of the epidermis, and decreased activity of sebaceous glands^3^. These effects are mainly mediated by sex hormones, particularly estrogen signaling, indicating that the endocrine pathways of hormones are important biological determinants of skin aging^4^.

In recent years, as the study of skin microbiota has progressed, an increasing number of reports have emerged on the association between aging and skin microbiota. In various ethnic groups, the alpha (α)-diversity of the facial skin microbiota in older adults is higher than that in younger individuals. Among Asian women, including Japanese women, a positive correlation was observed between *Cutibacterium acnes* (*C. acnes*) abundance and sebum content, and the average *C. acnes* abundance and sebum content on the foreheads of older women were lower than those of younger women^5–7^. In an observational study of Chinese individuals, the facial skin microbiota was divided into two types: *Moraxella osloensis* (*M. osloensis*)–dominated and *C. acnes*–dominated^5^. The proportion of older adults with the former type was higher than that of younger individuals. The high α-diversity of the skin microbiota observed in older or menopausal women is thought to be influenced by decreased sebum production and the associated decline in *C. acnes* abundance^8,9^. The fungus *Malassezia* is more prevalent on the faces of adults than on those of children. *Malassezia* and its lipid metabolites have been implicated in skin dryness and inflammation in adults^10^. However, how changes in the skin microbiota associated with decreased sebum secretion affect the process of skin aging and other skin properties is unclear.

In parallel, skincare research has explored the beneficial effects of skin microbiota. For example, in Indian individuals, multiple regression analysis demonstrated that *Streptococcus* and *Haemophilus* may influence stratum corneum water content^7^. In addition, the abundance of *Staphylococcus hominis* (*S. hominis*) has been reported to be higher on the cheeks of Japanese individuals with favorable skin properties, such as greater water content, lower transepidermal water loss (TEWL), and higher lightness. Application of *S. hominis* has also been shown to improve pore count, melanin index, and other parameters^11^. Although many reports have shown associations between skin microbiota and skin properties in specific populations, few have analyzed the relationship between specific bacterial species and skin characteristics within groups of the same ethnicity but differing by age or skin condition. Furthermore, little is known about how the relationship between skin properties and microbiota changes with aging in the same individual.

In this observational study, to clarify differences in the relationship between skin properties and microbiota under varying sebum levels and age groups, we conducted a subgroup analysis of Japanese women based on age, menopausal status, and sebum levels, which change significantly with aging.

## Results

### Skin properties and bacterial characteristics

Of the 322 enrolled participants, 5 were excluded based on the exclusion criteria (symptoms of atopic dermatitis, administration of antiallergic agents that may affect skin condition, or receipt of facial aesthetic treatment within 1 month of skin microbiota collection). The remaining 317 participants were included in this analysis. Correlation analyses among parameters across all participants showed that sebum content, porphyrins, and pores were positively correlated with one other, and all three were negatively correlated with age (Supplementary Fig. S1 online). In addition, common correlations were observed, such as the negative correlation between capacitance, a measure of skin water content, and transepidermal water loss (TEWL), as well as the positive correlations between age and various types of spots and color unevenness.

**Fig. 1:**
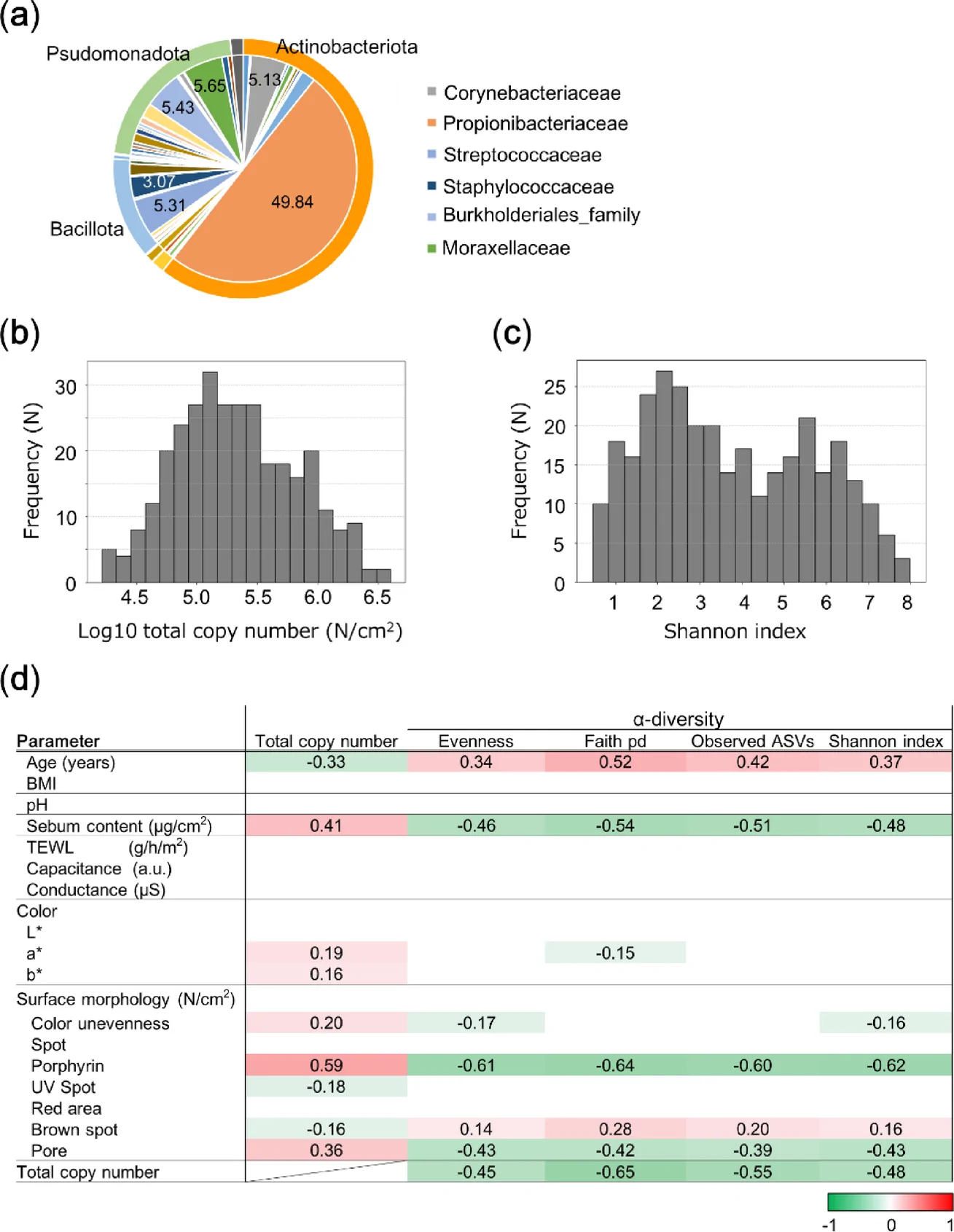
Characteristics of skin microbiota in study participants. (**a**) Average relative abundance of skin microbiota at the family level. The outer circle represents phylum-level composition, while the inner circle represents family-level composition. (**b**) Distribution of the total copy number, measured by qPCR, as 16 S rDNA gene copy numbers per cm^2^. (**c**) Distribution of the Shannon index. (**d**) Spearman’s rank correlation coefficients between microbiota indices and skin properties with significant differences (*p* < 0.05). Green indicates negative correlations; red indicates positive correlations. *Burkholderiales_family*, Burkholderiales-affiliated bacteria of unknown family; BMI, body mass index; TEWL, transepidermal water loss; L*, a*, b*, colorimetric parameters (lightness, red–green, and yellow–blue, respectively); UV, ultraviolet; pd, phylogenetic diversity; ASVs, amplicon sequence variants.

The skin microbiota composition at the family level revealed that　*Propionibacteriaceae* accounted for approximately half of the total abundance, and families with an average relative abundance greater than 3% included *Moraxellaceae* (5.65%), *Burkholderiales_family* (5.43%), *Streptococcaceae* (5.31%), *Corynebacteriaceae* (5.13%), and *Staphylococcaceae* (3.07%) (Fig. 1a). Absolute abundance (total copy number), calculated using 16 S ribosomal DNA, showed that some participants had up to 1.0 × 10^6^ copies/cm^2^ (Fig. 1b). However, most participants had fewer than 1.0 × 10^6^ copies/cm^2^, and half of the cohort had fewer than 2 × 10^5^ copies/cm^2^. The distribution of the Shannon index, a measure of bacterial α-diversity, was polarized, with one group exhibiting low diversity (index 0–4) and the other exhibiting high diversity (index 5–8) (Fig. 1c). Correlation analyses of total copy number, Shannon index, age, and skin properties revealed that total copy number was negatively correlated with age, but positively correlated with sebum content, porphyrin, and pores (Fig. 1d). In contrast, the Shannon index was positively correlated with age, but negatively correlated with sebum content, porphyrins, and pores.

### Age-related changes in skin properties and microbiota

**Fig. 2:**
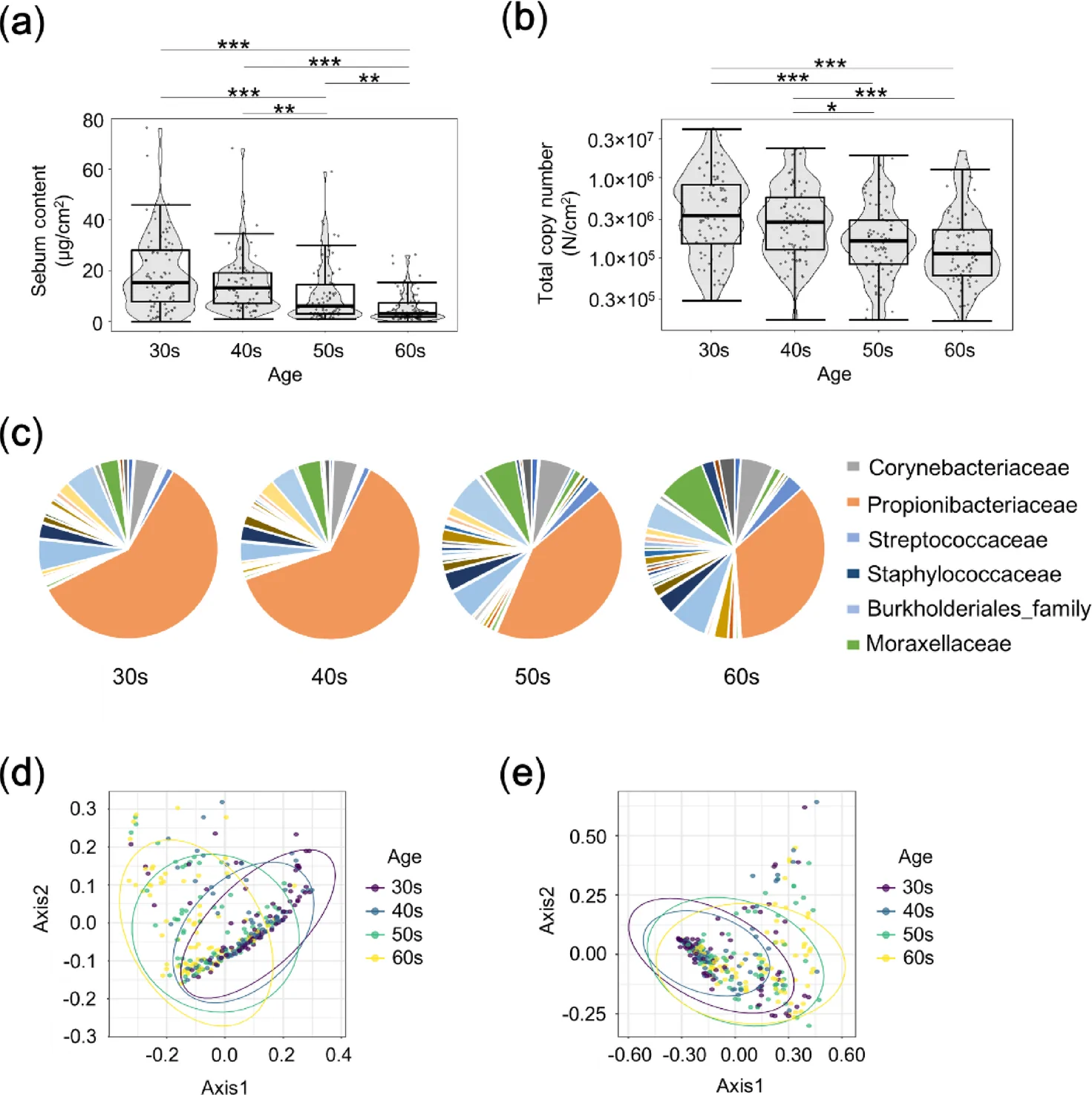
Age-related changes in skin properties and microbiota. (**a**) Violin plots with embedded box plots showing the distribution and median value of sebum content across age groups. (**b**) Violin plots with embedded box plots showing the distribution and median of total copy number. (**c**) Relative abundance of skin microbiota at the family level. (**d**) Principal coordinates analysis (PCoA) plots of unweighted UniFrac distances. (**e**) PCoA plots of weighted UniFrac distances. Statistical analyses between subgroups were performed using the Kruskal-Wallis test, followed by the Steel-Dwass post-hoc test. * *p* < 0.05, ** *p* < 0.01, *** *p* < 0.001.

Participants were subgrouped by age, and skin properties were compared across age groups. Sebum content, L^*^ value, porphyrins, and pores were lower in older age groups (Supplementary Table S2 online). In particular, sebum content showed pronounced age-related differences, with significant differences among all age groups except between those in their 30 s and 40 s (Fig. 2a).

Comparison of skin microbiota by age showed that total copy number was lower in older age groups, with significantly higher values in participants in their 30 s and 40 s compared with those in their 50 s and 60 s, respectively (Fig. 2b). Across age groups, microbiota composition shifted with increasing age, showing a lower abundance of *Propionibacteriaceae* and a higher abundance of *Moraxellaceae*, while the α-diversity peaked in individuals in their 60s. (Fig. 2c). The distribution of UniFrac distance, which measured the similarity of skin microbiota composition between groups, was comparable between the 30 s and 40 s and the 50 s and 60 s (Fig. 2d,e).

### Difference in the relationship between skin properties according to sebum content

**Fig. 3:**
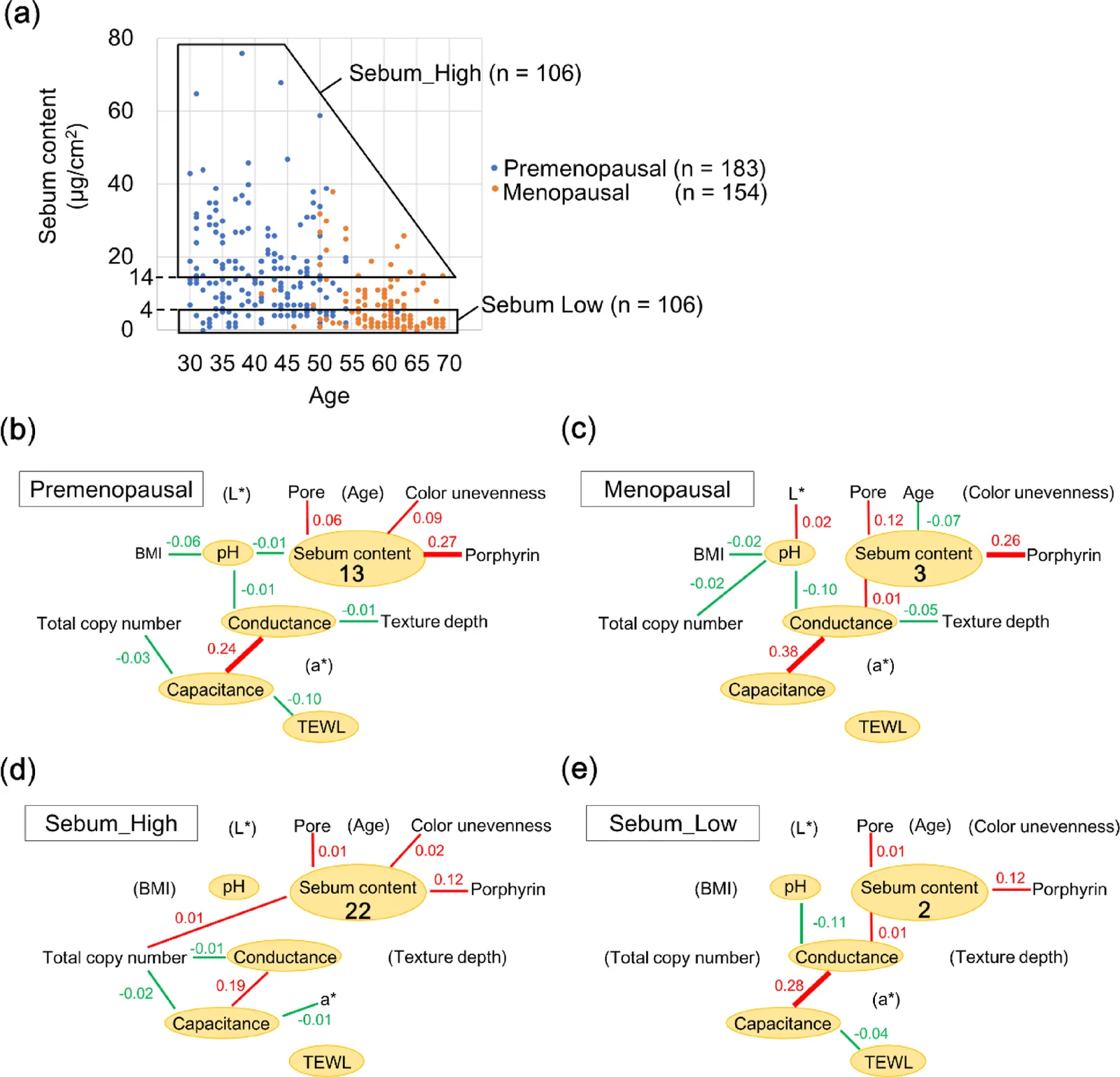
Relationships between skin properties according to the menopausal status and sebum content. (**a**) Menopausal status and distribution of sebum content in each subgroup. (**b–e**) Relationships between skin properties within each subgroup. The diagram shows only the factors directly connected to the five selected parameters, highlighted by yellow circles. The values inside the yellow circles represent the median sebum content. Red lines indicate significant positive associations, and green lines indicate significant negative associations. The numbers alongside the lines represent Graphical Lasso coefficients. TEWL, transepidermal water loss; BMI, body mass index; L*, a*, colorimetric parameters (lightness and red–green, respectively).

Sebum content is an important factor in the skin environment and changes with aging and menopause; therefore, participants were subgrouped according to sebum levels. The analysis was conducted using subgroups based on menopausal status (Analysis 1) and those obtained by dividing participants equally according to the ranked sebum content (Analysis 2) (Fig. 3a).

Analysis 1: Significant group differences were observed in age, pH, sebum content, TEWL, capacitance, L* value, and all surface morphology parameters (Supplementary Table S3 online). Graphical Lasso-based network analysis incorporating skin properties and total copy number revealed differences between groups in the associations between five parameters reflecting the basic physiological status of the skin—pH, sebum content, TEWL, capacitance, and conductance—and other parameters. The connection between sebum content and color unevenness, and between TEWL and capacitance were observed only in the Premenopausal group, whereas the connection between sebum content and conductance was present only in the Menopausal group (Fig. 3b,c). Furthermore, the Spearman correlation coefficients for the pairs differed significantly or showed a trend toward differences among the groups (Table 1). In contrast, other pairs that showed an association in either group did not exhibit significant differences in Spearman correlation coefficients between the groups.

**Table 1:**
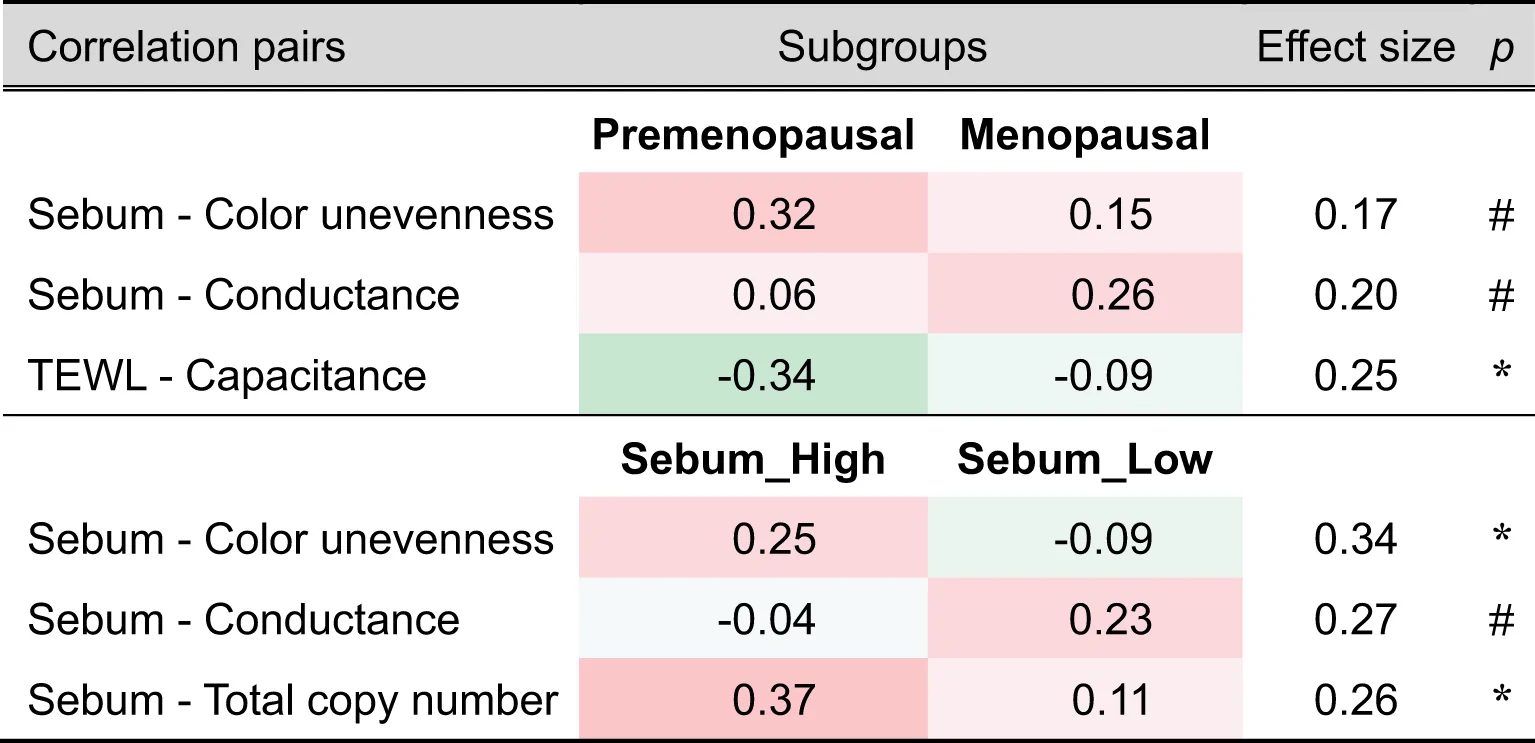
Group differences in the correlation between skin properties. The pairs of skin properties involving pH, sebum content, TEWL, capacitance, and conductance that showed statistically significant differences in correlation coefficients between the groups are summarized. Correlations were assessed using Spearman’s rank correlation coefficient, and differences between groups were evaluated using Fisher’s z-test after z-transformation. The color represents the direction and strength of the correlation (green indicates a negative correlation and red indicates a positive correlation). TEWL, transepidermal water loss; Effect size, absolute difference in correlation coefficients. # *p* < 0.1; * *p* < 0.05.

Analysis 2: Significant group differences were observed in age, sebum content, conductance, a* value, porphyrins, brown spots, and pores (Supplementary Table S4 online). Network analysis revealed that the connection between sebum content and color unevenness, and between sebum content and total copy number were observed only in the Sebum_High group, whereas the connection between sebum content and conductance was present only in the Sebum_Low group (Fig. 3d,e). Furthermore, the Spearman correlation coefficients for the pairs differed significantly or showed a trend toward differences among the groups (Table 1). The other pairs that showed an association in either group did not exhibit significant differences in Spearman correlation coefficients between the groups.

### Difference in the relationship between skin properties and microbiota according to sebum content

MaAsLin3 (Multivariate Association with Linear Models 3) was used for the absolute abundance data at the genus level, including the interaction terms to investigate the relationship between skin microbiota and pH, sebum content, TEWL, capacitance, and conductance. The models included interaction terms with sebum content (in all participants; Table 2) or with the group (Premenopausal vs. Menopausal and Sebum_High vs. Sebum_Low; Table 3).

**Table 2:** Genera significantly associated with pH, sebum content, TEWL, and capacitance in all participants.

Variable of interest	Genus (Response variable)	Prevalence (%)	coef	stderr	*p*	Interaction (× Sebum)	
						coef	*p*
pH	*Leptotrichia*	90.54	−0.62	0.14	**		
	*Capnocytophaga*	90.54	−0.61	0.16	**		
	*Neisseria*	98.74	−0.57	0.16	**		
	*Pseudopropionibacterium*	61.83	−0.56	0.15	**		
	*Streptococcus*	100.00	−0.43	0.13	*		
	*Abiotrophia*	78.86	−0.62	0.18	*		
	*Rothia*	99.05	−0.45	0.14	*		
	*Haemophilus*	96.85	−0.47	0.15	*		
	*Corynebacterium*	100.00	−0.37	0.11	*		
	*Gemella*	95.58	−0.46	0.15	*		
	*Aggregatibacter*	63.41	−0.49	0.17	*		
	*Bartonella*	65.30	−0.59	0.20	*		
	*Fusobacterium*	94.32	−0.43	0.15	*		
Sebum content	*Cutibacterium*	100.00	0.52	0.13	**		
	*Cloacibacterium*	91.17	−0.80	0.21	**		
	*Lawsonella*	97.79	0.76	0.21	*		
	*Kingella*	58.36	−0.49	0.15	*		
	*Bartonella*	65.30	0.78	0.23	*		
TEWL	*Leptotrichia*	90.54	−0.61	0.16	*	−0.75	*
	*Neisseria*	98.74	−0.58	0.17	*	−0.87	*
	*Kingella*	58.36	−0.50	0.13	**		
	*Lautropia*	82.02	−0.43	0.14	*		
Capacitance	*Chryseobacterium*	87.70	−0.59	0.19	*		

MaAsLin3 (Multivariate Association with Linear Models 3) was applied to absolute abundance data. The interaction term between the variable of interest and sebum content suggests that sebum content modifies the association between the variable of interest and skin microbiota. TEWL, transepidermal water loss; coef, regression coefficient; stderr, standard error of the coefficient; *p*, Benjamini–Hochberg adjusted *p*-value. * *p* < 0.05; ** *p* < 0.01.

**Table 3:** Genera significantly associated with pH, sebum content, TEWL, capacitance, and conductance in subgroups.

Variable of interest	Genus (Response variable)	Prevalence (%)	coef	stderr	*p*	Interaction (× Group)	
						coef	*p*
**Premenopausal group**							
pH	*Leptotrichia*	90.54	−0.81	0.22	*		
Sebum content	*Cloacibacterium*	86.89	−0.91	0.24	*		
	*Cutibacterium*	100.00	0.51	0.15	#		
	*Lawsonella*	97.79	1.66	0.46	#		
TEWL	*Lautropia*	79.78	−0.65	0.18	*		
	*Janibacter*	60.66	0.83	0.24	*		
	*Kingella*	51.91	−0.59	0.17	*		
Capacitance	*Lactococcus*	61.20	−1.49	0.32	**	−0.90	**
	*Acetobacteraceae_g*	91.17	1.59	0.47	#		
Conductance	*Lactococcus*	61.20	−1.07	0.33	#	−0.49	#
**Menopausal group**							
Sebum content	*Lawsonella*	97.76	1.10	0.30	#		
**Sebum_High group**							
TEWL	*Neisseria*	83.96	−1.46	0.39	*	−0.94	*
	*Leptotrichia*	97.17	−1.33	0.35	*	−0.72	*
	*Kingella*	47.17	−0.87	0.26	*		
	*Granulicatella*	92.45	−0.81	0.25	#		
	*Lautropia*	77.36	−0.80	0.25	#		
	*Streptococcus*	100.00	−0.63	0.22	#		

MaAsLin3 (Multivariate Association with Linear Models 3) was applied to absolute abundance data. The interaction term between the variable of interest and sebum content suggests that sebum content modifies the association between the variable of interest and skin microbiota. TEWL, transepidermal water loss; coef, regression coefficient; stderr, standard error of the coefficient; *p*, Benjamini–Hochberg adjusted *p*-value. * *p* < 0.05; ** *p* < 0.01.

In all participants, 13 genera were significantly associated with pH, with *Leptotrichia* having the highest regression coefficients (Table 2). Five genera were significantly associated with sebum content, with *Cloatibacterium* having the highest regression coefficients. Four genera were significantly associated with TEWL, with *Leptotrichia* having the highest regression coefficients. The negative associations of *Leptotrichia* and *Neisseria* with TEWL were significantly influenced by sebum content, becoming stronger at higher sebum levels. *Chryseobacterium* was significantly associated with capacitance.

In the subgroups based on menopausal status, genera that showed significant associations with skin properties were observed only in the Premenopausal group (Table 3). *Leptotrichia* and *Cloacibacterium* were significantly associated with pH and sebum content, respectively. Three genera were significantly associated with TEWL, with *Lautropia* having the highest regression coefficients. *Lactococcus* was significantly associated with capacitance, and this negative association was significantly influenced by the group.

In the subgroups based on sebum content, genera showing significant associations with skin properties were observed only in the Sebum_High group (Table 3). Three genera were significantly associated with TEWL, with *Neisseria* and *Leptotrichia* showing high regression coefficients, and their negative association with TEWL was significantly influenced by the group.

## Discussion

In this study, we analyzed the relationship between facial skin properties and microbiota on the foreheads of Japanese women, focusing on the differences in sebum content that occur with aging and menopause. The main findings are as follows. First, subgroup analyses based on menopausal status and sebum content suggested that the relationships between skin properties and between skin properties and bacterial genera varied according to sebum levels. Second, associations between specific bacterial genera and skin properties, mainly pH, TEWL, and hydration, were observed primarily in participants with relatively preserved sebum levels.

Sebum, a major component of the hydrolipid film covering the skin surface, regulates TEWL and stratum corneum hydration, thereby supporting epidermal barrier function^12,13^. It is also a fundamental component of the microbial network on the skin surface, serving as a nutrient source that supports the interactions of multiple microbial species^14,15^.

Against this background, the differences observed in this study, both in the relationships among skin properties and in the associations between skin properties and microbiota across groups with different sebum levels, are suggestive. In the analysis of relationships among skin properties, the associations between sebum content and color unevenness, sebum content and conductance, and TEWL and capacitance differed between the groups with varying sebum levels (Fig. 3; Table 1). These findings indicate that sebum level and menopausal status are associated with differences in the relationship between skin properties. In groups with low sebum levels, a positive correlation was observed between sebum content and conductance, whereas in groups with high sebum levels, a positive correlation was observed between sebum content and color unevenness. Given that sebum is susceptible to oxidation and that oxidized lipids can exert irritant effects on the skin^13,16^, it would be worthwhile to evaluate whether appropriate control of both the quantity and quality of sebum contributes to improvements in hydration and color unevenness in future studies.

Differences in sebum levels were also reflected in skin microbiota composition. The bimodal distribution of the α-diversity index and age-group comparison of skin microbiota showed that participants in their 50–60 s predominantly had skin microbiota with higher α-diversity, whereas those in their 30–40 s more often had microbiota with lower α-diversity and a higher abundance of Propionibacteriaceae (Figs. 1c and 2b,d). Notably, the two microbiota profiles identified as bimodal patterns in this study showed similarity to the two major skin microbiota cutotypes described in a Chinese cohort^5^. Our findings are also partly consistent with those of a recent pilot study^17^ that compared premenopausal and postmenopausal women and found lower *Cutibacterium* abundance and higher bacterial diversity in the Menopausal group.

Estrogens and androgens are known to regulate sebaceous gland activity, sebum production, barrier function, hydration, and wound repair^3,4,12^. Therefore, menopausal changes in sex hormone levels may alter the facial skin environment, including sebum content and other related biophysical properties. Direct evidence that sex hormones broadly regulate commensal skin bacteria remains limited, making indirect pathways through host skin physiology more plausible. As this study did not include measurements of circulating sex hormone levels, any conclusions regarding the role of sex hormone-mediated mechanisms are speculative.

In line with these observations, several genera were significantly associated with pH, sebum content, TEWL, and capacitance, and some of these associations were observed only in the Premenopausal or Sebum_High subgroups (Tables 2 and 3). Moreover, a few associations showed significant interaction terms with sebum content, suggesting that these relationships differed according to sebum levels. The absence of significant associations in groups with lower sebum levels may reflect lower copy numbers, reduced statistical power, or unmeasured age-related skin factors, although further investigation is required to validate this interpretation. Because low bacterial abundance increases technical variability in 16 S rDNA sequencing and qPCR^18^, more refined analytical approaches may be needed to elucidate skin property–microbiota relationships in older adults or low sebum groups.

When focusing on individual bacterial genera, *Streptococcus* was identified as a genus associated with pH and TEWL in all participants and those of the Sebum_High subgroup. A study based on multiple regression analysis reported that *Streptococcus* positively affected forehead hydration in Indian participants^7^. Moreover, culture supernatants of skin-isolated *Streptococcus* restored skin barrier function, emphasizing the importance of polyamine production^19^. Polyamines are one of the antioxidants and promote the expression of barrier-related and lipid synthesis–related genes in epidermal keratinocytes^19,20^. In this study, *Streptococcus* exhibited the second-highest relative abundance, highlighting the need for further studies on *Streptococcus* species and their contributions to barrier function.

Other genera associated with pH, TEWL, and capacitance included *Leptotrichia*, *Neisseria*, *Aggregatibacter*, *Kingella*, *Lautropia*, and *Chryseobacterium*, which are known oral or environmental microbiota detected consistently across participants (Tables 2 and 3). Although contamination cannot be completely excluded, these genera showed high detection rates (Table 2), and considering the timing and method of sampling, we interpreted this as colonization rather than contamination. This is supported by previous studies that analyzed the V1–V2 region of the forehead microbiota in Japanese women, which also detected oral genera^6^.

The mechanisms underlying these associations remain unclear, but two hypotheses can be considered: (i) the coexistence of diverse microbes may contribute to the maintenance of skin homeostasis and (ii) specific microbial components or metabolites may directly affect skin physiology. Regarding the former, similar findings have been reported in Asian women^21^. In addition, we observed a trend toward a negative correlation between TEWL and the number of detected genera (observed ASVs), with participants showing moderate sebum levels and high observed features that exhibited low TEWL (Supplementary Fig. S2 online).

With respect to the latter hypothesis, although the average relative abundance of *C. acnes* was exceptionally high on the forehead of the study participants, its association was observed only with sebum levels (Table 2), and no associations with other skin properties were noted. Under conditions of sufficient sebum availability, multiple metabolic processes occur concurrently, including the hydrolysis of sebum triglycerides by *C. acnes* lipases, release of free fatty acids, and subsequent secondary metabolism by other bacterial species^14,15^. Free fatty acids may contribute to skin homeostasis by helping maintain an acidic skin surface and supporting antimicrobial defense and barrier-related processes^22,23^. Such metabolic interactions between sebum and multiple bacterial genera may alter the skin surface environment and influence skin properties, which could explain the observed associations between skin properties and multiple bacterial genera in groups with higher sebum levels.

These insights may generate hypotheses for future skincare strategies: for high sebum levels, controlling excess sebum and using emollients that enhance microbial diversity could help improve barrier function. Additionally, mitigating the negative effects of sebum, such as increased surface roughness resulting from the accumulation of oxidized lipids, may be important. In cases of low sebum levels, such as those associated with menopause or constitutional factors, emollients that help compensate for the protective functions of sebum may be worth considering. Furthermore, approaches that limit the excessive dominance of lipid-preferring bacteria, such as *C. acnes*, while selectively promoting beneficial microbiota, may be effective.

However, this study has several limitations, and further investigation is required to achieve a more comprehensive understanding of the relationships among sebum, skin microbiota, and skin properties. First, the study included only Japanese women, limiting the generalizability of the findings to other genders, ethnicities, or cultural backgrounds. Second, because the study focused exclusively on the forehead, the observed relationships among skin properties, sebum, and microbiota may not apply to other facial areas. Third, as a cross-sectional observational study, the results may have been influenced by measurement methods and sampling timing. Microbial samples were self-collected by the participants, which may have introduced inter-individual variability in sampling efficiency. In addition, skin properties were measured at a single time point after facial cleansing and acclimation; therefore, these measurements may not fully reflect participants’ average or long-term skin conditions.

Fourth, only the total sebum content was assessed, and the relationships between individual sebum components and skin microbiota remain unclear. On the forehead, triglycerides, wax esters, and squalene are recognized as the major sebum components^24^; however, these lipid classes differ in their susceptibility to bacterial utilization and oxidative modification^25,26^. Thus, future studies should obtain quantitative data on individual sebum components to investigate their specific associations with skin properties and microbiota.

Fifth, the taxonomic classification of skin microbiota may be affected by biases related to the sampling method and choice of the amplified genetic region^27^. As adhesive patches were used for sample collection, this sampling approach may fail to adequately capture microbiota residing in deeper, more anaerobic regions of the hair follicles. Among the genera listed in Tables 2 and 3, *Leptotrichia* was the only strictly anaerobic genus, whereas most other genera were capable of surviving under aerobic conditions. This suggests that the present analysis primarily reflects microbial relationships in aerobic surface skin environments.

Lastly, the influence of nonbacterial microorganisms such as fungi was not evaluated. Recent studies have reported a positive correlation between oxidized lipids and increased abundance of *Malassezia restricta* in adult skin, suggesting that the combination of sebum oxidation and *M. restricta* proliferation can exacerbate skin inflammation^10^. To comprehensively understand skin conditions associated with high sebum levels, the entire microbial network associated with sebum, including bacterial and fungal communities, must be considered.

Given these limitations, future longitudinal studies integrating spatially resolved sampling of the skin surface and deeper hair follicle regions, simultaneous measurements of sebum quantity and composition, and analyses of bacterial metabolic functions using metagenomic and metabolomic approaches are expected to provide a more detailed understanding of the relationship between skin properties and microbiota.

## Conclusion

The findings of this study suggest that sebum may play an important role in modulating the association between skin properties and microbiota. From a practical perspective, these findings may help inform skincare strategies that consider both sebum levels and microbiota-related characteristics, potentially contributing to more personalized approaches across different life stages.

## Methods

### Participants

This study was conducted in accordance with the Declaration of Helsinki, and the study protocol was approved by the Human Study Ethics Committee of Shiba Palace Clinic (Tokyo, Japan; Approval number 147309 − 30486, accepted on October 14, 2021). A total of 322 healthy Japanese women aged 30–69 years were recruited by SOUKEN Inc. (Tokyo, Japan), with approximately 80 participants in each age group from the 30 s to the 60 s (Supplementary Fig. S2 online). All participants provided written informed consent prior to their participation.

After confirming their intention to participate, a self-administered questionnaire was administered regarding age, medication status, menopausal status, and lifestyle habits. These data were used to determine exclusion before analysis and to create subgroups based on age and menopausal status of participants.

### Study period and seasonal context

This study was conducted during the winter. Sample collection and clinical skin parameter measurements were performed between November 25 and December 9, and DNA extraction and sequencing of skin microbiota were performed in December.

### Sample collection

**Fig. 4:**
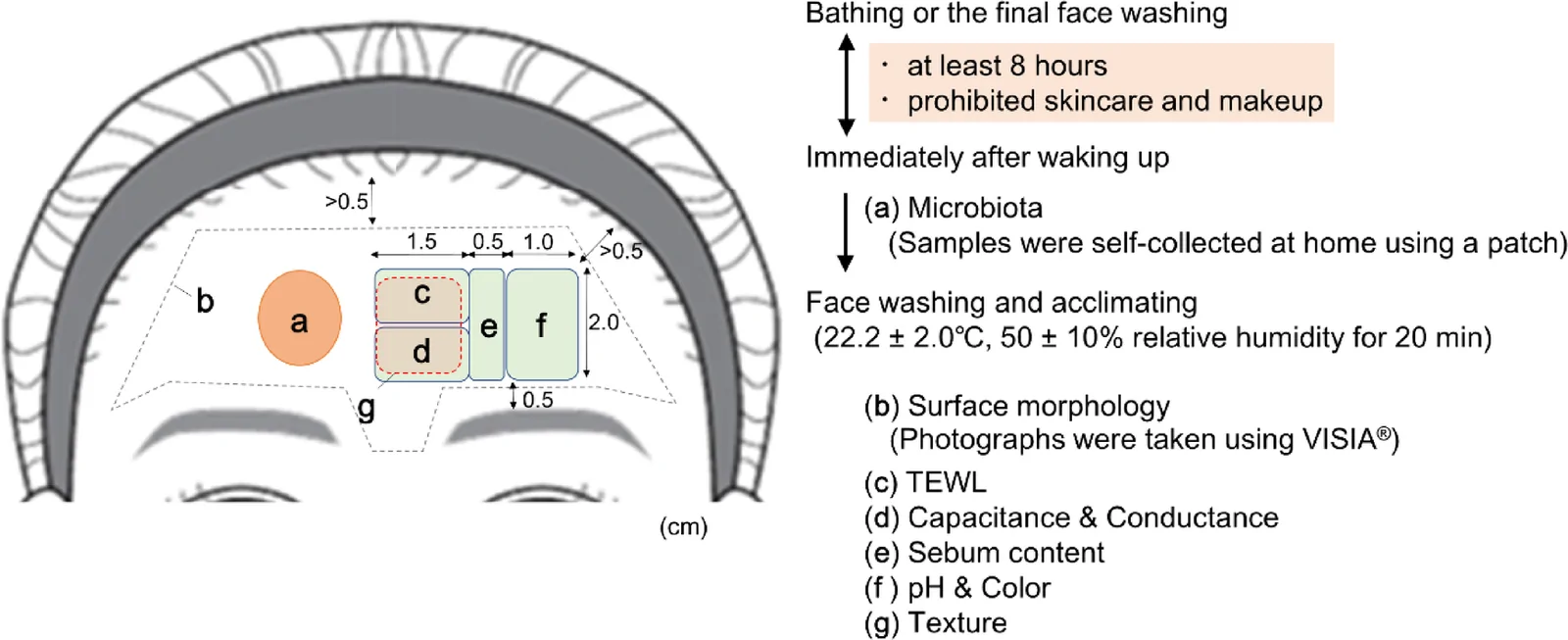
Overview of skin microbiota collection and skin property measurements. Various restrictions and the sites of sampling and measurement are indicated. The measurements were conducted in alphabetical order. TEWL, transepidermal water loss.

Participants were instructed to self-collect skin samples and wait at least 8 h between washing their face the day before and collecting the samples the next morning (Fig. 4). All skincare products, including the use of moisturizers and makeup, were prohibited during this period. Upon waking and before washing their face, participants put on disposable gloves and applied a sampling patch (MySkin^®^, an oval-shaped adhesive tape, 300 mm^2^) above the right eyebrow on the forehead. After 1 min, the patch was removed from the forehead and mounted on a special holder. The participants stored the patches and skin samples at room temperature and submitted them to SOUKEN Inc. within the same day (within several hours). Subsequently, the patches were maintained at −20 °C by SOUKEN Inc. and transported in a frozen state to MySkin Co., Ltd. (Tokyo, Japan), where skin microbiota analysis was performed.

### DNA extraction, amplification of the 16 S rRNA gene region, and next-generation sequencing

Skin microbiota analysis was performed by MySkin Co., Ltd. DNA was extracted as previously described^28^. After performing quality control, the V1–V2 region of the 16 S rRNA gene in diluted DNA samples was amplified using the 27Fmod forward and 338R reverse primers, which contained Golay barcodes and Illumina adapter sequences. DNA concentration in each sample was measured, and all samples were normalized to the same concentration to prepare the sequencing library. Libraries were sequenced using 300-bp paired-end reads on the MiSeq platform (Illumina, USA).

Negative controls (blank patches and reagent controls) were validated prior to the study using the same workflow. Sequencing of negative controls yielded exactly 2,657 reads, whereas the sample libraries were adjusted to yield ≥ 50,000 reads.

### Taxonomic assignment of skin microbiota

Amplicon sequence reads were processed using QIIME 2 (version 2018)^29^. Raw amplicon sequencing data were processed into a table of exact amplicon sequence variants (ASVs) present in each sample using the DADA2 plugin^30^. PhiX reads and chimeric sequences were filtered using the pooled consensus method. The resulting feature table was used for taxonomic assignment based on the SILVA_138 database by training a Naïve Bayes classifier using the q2-feature-classifier plugin^31^. Absolute count data from taxonomic assignments were normalized to relative abundance at the phylum, family, and genus levels using the taxa plugin. *De novo* multiple sequence alignment was performed using the MAFFT method and filtered to remove highly variable regions^32^. Sequences were clustered into operational taxonomic units based on 97% sequence similarity. α-diversity of the samples were analyzed at a sampling depth of 5,000, retaining 317 samples. Shannon’s diversity index, observed ASVs, evenness, and Faith’s phylogenetic diversity indices were calculated using the diversity plugin in QIIME 2. For beta-diversity analysis, weighted and unweighted UniFrac distances were calculated using the capscale function in the R package vegan^33^, followed by visualization via principal coordinate analysis (PCoA).

### Quantification of the total 16 S rDNA copy number of skin microbiota

The total 16 S rDNA copy number of skin microbiota was determined by quantitative real-time polymerase chain reaction (qPCR) using the forward 27modF (5′-GRGTTTGATYMTGGCTCAG-3′) and reverse 338R (5′-TGCTGCCTCCCGTAGGAGT-3′) primers on a StepOne Plus system (Applied Biosystems, USA). KAPA SYBR Fast qPCR Universal (KAPA Biosystems, USA) was used for PCR reactions. PCR conditions were: initial denaturation at 95 °C for 20 s, followed by 40 cycles of 95 °C for 3 s and 60 °C for 30 s, followed by a standard denaturation curve protocol. Synthetic DNA based on the 16 S rRNA gene of *C. acnes* was used as the standard. The detection limit for total copy number was approximately 5,000 cells.

### Clinical skin parameter measurement

Skin property parameters were measured on the same day as skin sample collection. After washing their faces under the same conditions, the participants acclimated in a room maintained at 22.0 ± 2.0 °C and 50 ± 10% relative humidity for 20 min. Measurements were then performed as described (Fig. 4, Supplementary Table S1 (online)). Sebum content was measured using a Sebumeter^®^ SM815 (Courage + Khazaka electronic GmbH, Germany). TEWL was measured using a Tewameter^®^ TM-300 (Courage + Khazaka electronic GmbH, Germany). Sebum content and TEWL were measured only once. pH was measured using MPA580 (Courage + Khazaka electronic GmbH, Germany) and the skin color was measured using a Spectrophotometer CM700d (Konica Minolta, Japan), reporting L*, a*, and b* values. The pH and skin color were measured twice, and average values were calculated. Skin moisture was measured using Corneometer^®^ CM825 (Courage + Khazaka Electronic GmbH, Germany) and SKICON-200EX (YAYOI, Japan). Measurements were performed five times, excluding the highest and lowest values, and the average was calculated. The number, volume ratio and depth of the skin texture were measured using silicon replicas and ASA-03R (ASCH JAPAN Co., Ltd., Japan). The parameters for skin surface morphology, specifically color unevenness, spots, porphyrins, UV spots, red area, blown spots, and pores, were measured using VISIA-Generation^®^ 7 − 2 (Canfield Scientific, USA).

### Criteria for subgrouping

Subgroups were defined as follows: Age: decade at the time of skin measurement (30s: *n* = 80; 40s: *n* = 78; 50s: *n* = 79; 60s: *n* = 80). Menopausal status: self-reported status approximately one month before measurement (Premenopausal: *n* = 183; Menopausal: *n* = 134). Sebum content: ranked sebum values were evenly divided into three groups (Sebum_Low: *n* = 106; Sebum_High: *n* = 106; and intermediate: *n* = 105) (Fig. 3a and Supplementary Tables S2–S4).

### Statistical analysis

All analyses were performed in R (version 4.5.2). Sebum values < 1 were excluded from all analyses for being below the reliable measurement range of the Sebumeter^®^ SM815. Age, body mass index, skin properties, and skin microbiota data were compared using the Kruskal–Wallis test for comparisons involving three or four groups (followed by the Steel–Dwass post hoc test) or the Mann–Whitney *U* test for two-group comparisons. Correlations were assessed using Spearman’s rank correlation coefficient. Differences in correlation coefficients between groups were evaluated using Fisher’s z test, which compares two independent correlation coefficients after Fisher’s z transformation and calculates a two-tailed *p*-value based on the standard error of the difference. Statistical significance was defined as *p* < 0.05.

### Graphical Lasso-based network analysis

Conditional dependencies among skin properties and total copy number were inferred using a Graphical Lasso approach. All variables were standardized to a zero mean and unit variance prior to the analysis. Network estimation was performed using the huge (version 1.3.5)^34^, and precision matrices were computed using glasso. Model selection employed the Stability Approach to Regularization Selection (STARS) with a threshold of 0.20. The optimal regularization parameter identified by STARS was applied to estimate the precision matrix, which was subsequently symmetrized to correct the numerical inconsistencies.

### Multivariate association with linear models

The associations between bacterial genera and skin properties were assessed using MaAsLin3 (version 1.1.2)^35^. Analyses were conducted on total copy number without normalization, applying log transformation to reduce skewness. Continuous covariates were standardized to a zero mean and unit variance to facilitate the comparison of coefficients. The model formula included an interaction term between the variable of interest and either sebum content or categorical group variables (e.g., menopausal status or sebum level). For analyses of all participants, age and parameters significantly correlated with the variable of interest were included as covariates. For subgroup analyses, the covariates comprised parameters correlated significantly with the variable of interest and those that showed significant differences among groups. Genera present in fewer than 10% of the samples were excluded prior to model fitting. *p*-values were adjusted for multiple testing using the Benjamini–Hochberg method, and results were filtered using FDR < 0.05.

## Supplementary Information

Below is the link to the electronic supplementary material.

Supplementary Material 1

## Data Availability

16 S rRNA gene amplicon sequencing data were deposited in the Sequence Read Archive of the National Center for Biotechnology Information under the accession number PRJNA1336993. Other datasets generated during and/or analyzed during the current study are available from the corresponding author upon reasonable request.

## Code availability

No custom scripts or bespoke computational tools were developed in this study. All analyses were conducted using previously published R packages, which are cited in the Methods Section.
