# Supplementary material for: Sebum levels are associated with the relationship between skin properties and microbiota in Japanese women
Source: Sci Rep. 2026 Jul 3;16:27681. doi: 10.1038/s41598-026-59379-w (PMC13542196; doi:10.1038/s41598-026-59379-w)

Supplemental data

Sebum Levels Are Associated with the Relationship Between Skin Properties and Microbiota in Japanese Women

Yuki Ishii, Miyuki Kudo, Akemi Imaoka, and Tatsuichiro Shima

Table S1 Skin properties measured in this study

Parameter	Unit	Reference
pH	-	-
Sebum content	μg/cm^2	-
TEWL	g/h/m^2	Trans epidermal water loss.
Capacitance	a.u.	Water content in the stratum corneum and the upper layer of the epidermis (approximately 15 μm from the surface). Reflects the water content deeper than Conductance.
Conductance	μS	Water content in the upper layer of the stratum corneum.
Color		
L*	-	The lightness value.
a*	-	Green indicates negative values and magenta indicates positive values.
b*	-	Blue indicates negative values and yellow indicates positive values.
Texture		
Number	N/mm	Number of textures per 1 mm straight line.
Volume ratio	μm^3/mm^2/100	Number of texture volume per millimeter square divided by 100.
Depth	μm	Average depth of texture.
Surface morphology		
Color unevenness	N/cm^2	Number of areas that differ in color from their surroundings. (Displayed as "Texture" on the instrument).
Spots	N/cm^2	
Porphyrins	N/cm^2	
UV Spots	N/cm^2	Number of potential spots that are not visible on the surface.
Red areas	N/cm^2	Number of areas with strong hemoglobin pigment.
Brown spots	N/cm^2	Number of spots with dark melanin pigment.
Pores	N/cm^2	

Figure S1. Relationship between physical and skin property parameters.

Spearman's rank correlation coefficients are shown; correlations with Benjamini–Hochberg adjusted *p*-values less than 0.05 are indicated. The color scale represents the direction and strength of the correlation (green indicates negative correlation, while red indicates positive correlation).

Abbreviations: BMI, body mass index; TEWL, transepidermal water loss; L*, a*, b*, colorimetric parameters (lightness, red–green, and yellow–blue, respectively).

Category	Parameter	Age	BMI	pH	Sebum content	TEWL	Capacitance	Conductance	L*	a*	b*	Texture Number	Texture Volume ratio	Texture Depth	Color unevenness	Spots	Porphyrins	UV Spots	Red areas	Brown spots	Pores
	Age				-0.49		-0.12		-0.22						0.36	0.44	-0.41	0.27	0.18	0.58	-0.17
	BMI			-0.20		0.17			-0.15						0.15	0.12			0.18		
	pH							-0.29	0.20												
	Sebum content							0.14		0.17							0.64			-0.27	0.42
	TEWL						-0.22								0.12			-0.14			0.13
Moisture	Capacitance							0.53		-0.16						-0.11				-0.13	
	Conductance													-0.16							
	L*									-0.38	-0.39				-0.50	-0.52		0.33	-0.15	-0.16	
Color	a*										0.40				0.32	0.25	0.18	-0.25			0.16
	b*														0.29	0.25	0.13	-0.32			
	Number												0.95	-0.40							
Texture	Volume ratio													-0.18							
	Depth																	-0.18		-0.13	
	Color unevenness															0.68	0.21		0.29	0.40	0.46
	Spots																		0.43	0.71	0.14
Surface morphology	Porphyrins																			-0.18	0.50
	UV Spots																			0.39	
	Red areas																			0.32	0.12
	Brown spots																				
	Pores																				

-1 0 1

Table S2. Demographic and clinical characteristics of groups based on age.

	30s (n=80)		40s (n=78)		50s (n=79)		60s (n=80)		
Parameter	Median	(IQR)	Median	(IQR)	Median	(IQR)	Median	(IQR)	*p*
Age (years)	34.50	(32.00–37.00)	44.50	(43.00–47.00)	54.00	(50.50–56.50)	63.00	(61.00–65.00)	***
BMI	19.98	(18.69–22.63)	20.62	(19.25–22.62)	20.69	(19.24–23.14)	21.08	(18.89–23.51)	
pH	6.38	(5.78–6.74)	6.45	(6.00–6.90)	6.20	(5.89–6.79)	6.13	(5.55–6.62)	
Sebum content (μg/cm^2)	15.00	(7.75–28.00)	13.00	(7.00–19.00)	6.00	(3.00–14.50)	3.00	(1.75–7.25)	***
TEWL (g/h/m^2)	11.05	(8.40–15.45)	12.60	(10.27–15.53)	10.70	(8.50–14.35)	10.40	(8.78–11.72)	*
Capacitance (a.u.)	38.00	(29.48–47.35)	37.25	(24.12–44.30)	36.60	(27.80–46.10)	32.40	(23.55–41.02)	*
Conductance (μS)	224.00	(178.50–288.25)	211.50	(174.00–262.50)	231.00	(158.00–300.50)	212.00	(154.25–257.25)	
Color									
L*	59.20	(57.58–60.60)	58.90	(57.08–60.68)	58.20	(56.10–59.15)	57.70	(56.20–59.35)	**
a*	5.51	(4.40–6.55)	5.63	(4.53–6.79)	5.39	(4.23–6.58)	5.42	(4.62–6.66)	
b*	17.10	(15.80–18.42)	17.00	(15.95–18.60)	17.40	(15.95–18.70)	17.40	(15.93–18.95)	
Texture									
Number (N/mm)	0.70	(0.36–1.07)	0.59	(0.35–0.95)	0.71	(0.44–1.04)	0.83	(0.42–1.10)	
Volume ratio (μm^3/mm^2/100)	24.50	(13.50–34.38)	19.70	(12.10–28.68)	24.20	(15.20–32.30)	26.35	(14.58–35.52)	
Depth (μm)	57.60	(55.65–59.95)	57.10	(55.23–59.08)	57.10	(54.90–58.85)	56.65	(55.20–59.45)	
Surface morphology (N/cm^2)									
Color unevenness	14.44	(6.00–19.98)	20.70	(11.05–27.89)	25.02	(14.71–36.30)	25.82	(16.00–42.30)	***
Spots	0.58	(0.40–0.81)	0.87	(0.68–1.15)	1.16	(0.82–1.46)	1.15	(0.77–1.46)	***
Porphyrins	46.20	(16.89–67.83)	36.80	(15.58–62.77)	15.88	(6.16–38.75)	11.96	(4.55–20.17)	***
UV Spots	5.24	(3.70–6.14)	5.91	(5.12–6.38)	6.04	(5.53–6.57)	6.04	(5.35–6.56)	***
Red areas	0.04	(0.00–0.09)	0.04	(0.03–0.10)	0.06	(0.03–0.10)	0.08	(0.04–0.14)	*
Brown spots	0.69	(0.49–1.04)	1.25	(0.90–1.68)	1.73	(1.12–2.60)	2.05	(1.54–2.76)	***
Pores	25.13	(17.78–32.05)	27.30	(18.14–33.20)	21.65	(13.09–29.30)	21.69	(13.56–28.43)	*
Total copy number (N/cm^2)	333536.03	(150500.87–819334.20)	274229.24	(126710.06–566394.52)	161992.07	(83369.43–293251.29)	111837.74	(60518.11–222457.01)	***
α-diversity									
Evenness	0.36	(0.23–0.50)	0.34	(0.23–0.48)	0.51	(0.36–0.66)	0.58	(0.43–0.70)	***
Faith pd	28.87	(21.70–34.29)	32.20	(26.56–38.30)	41.39	(31.43–49.88)	47.27	(39.67–57.03)	***
Observed ASVs	207.00	(121.50–301.25)	229.00	(163.00–298.00)	319.00	(211.00–453.50)	400.50	(266.00–496.50)	***
Shannon index	2.63	(1.64–4.17)	2.58	(1.77–3.89)	4.10	(2.80–5.73)	5.12	(3.60–6.28)	***

Subgrouping was based on age on the day of skin measurement. Values are presented as median and IQR. The total copy number was measured by qPCR, as 16S rDNA gene copy numbers per cm^2. Statistical analyses between subgroups were performed using the Kruskal–Wallis test.

Abbreviations: BMI, body mass index; TEWL, transepidermal water loss; L*, a*, b*, colorimetric parameters (lightness, red–green, and yellow–blue, respectively); UV, ultraviolet; pd, phylogenetic diversity; ASVs, amplicon sequence variants. * $p < 0.05$, ** $p < 0.01$, *** $p < 0.001$.

Table S3. Demographic and clinical characteristics of groups based on menopausal status.

Parameter	Premenopausal (n=183) Median	(IQR)	Menopausal (n=134) Median	(IQR)	*p*
Age (years)	41.00	(35.00–47.50)	61.00	(56.00–63.00)	***
BMI	20.32	(19.06–22.56)	21.27	(19.02–23.52)	
pH	6.40	(5.94–6.81)	6.14	(5.71–6.65)	*
Sebum content (μg/cm^2)	13.00	(6.00–21.00)	3.00	(2.00–9.75)	***
TEWL (g/h/m^2)	11.60	(8.95–15.50)	10.50	(8.83–12.55)	*
Capacitance (a.u.)	37.60	(29.20–45.75)	33.95	(23.60–44.08)	*
Conductance (μS)	221.00	(177.00–279.50)	221.00	(156.00–283.25)	
Color					
L*	58.90	(57.10–60.55)	58.00	(56.23–59.40)	**
a*	5.75	(4.50–6.73)	5.37	(4.47–6.66)	
b*	17.20	(15.90–18.80)	17.40	(15.62–18.60)	
Texture					
Number (N/mm)	0.65	(0.36–1.05)	0.74	(0.40–1.10)	
Volume ratio (μm^3/mm^2/100)	22.30	(13.60–32.85)	25.25	(14.03–34.30)	
Depth (μm)	57.30	(55.30–59.60)	57.00	(55.12–59.18)	
Surface morphology (N/cm^2)					
Color unevenness	18.16	(9.38–27.23)	24.29	(14.89–37.48)	***
Spots	0.78	(0.48–1.08)	1.12	(0.74–1.45)	***
Porphyrins	38.74	(16.02–65.97)	11.12	(4.87–21.23)	***
UV Spots	5.70	(4.83–6.30)	6.06	(5.42–6.51)	***
Red areas	0.04	(0.03–0.10)	0.07	(0.03–0.13)	*
Brown spots	1.03	(0.64–1.53)	1.94	(1.35–2.73)	***
Pores	25.43	(16.68–33.04)	21.57	(13.32–29.06)	**
Total copy number (N/cm^2)	291493.41	(125582.87 –715569.70)	128051.39	(68224.10 –222020.28)	***
α-diversity					
Evenness	0.35	(0.24–0.53)	0.55	(0.42–0.70)	***
Faith pd	30.71	(23.78–38.66)	45.48	(36.15–53.59)	***
Observed ASVs	228.00	(144.00–323.50)	376.50	(250.25–476.50)	***
Shannon index	2.71	(1.75–4.29)	4.70	(3.36–6.18)	***

Subgrouping was based on menopausal status (self-reported via questionnaire) approximately one month before the day of skin measurements. Values are presented as median and IQR. The total copy number was measured by qPCR, as 16S rDNA gene copy numbers per cm^2. Statistical analyses between subgroups were performed using the Mann–Whitney *U* test.

Abbreviations: BMI, body mass index; TEWL, transepidermal water loss; L*, a*, b*, colorimetric parameters (lightness, red–green, and yellow–blue, respectively); UV, ultraviolet; pd, phylogenetic diversity; ASVs, amplicon sequence variants. * $p < 0.05$, ** $p < 0.01$, *** $p < 0.001$.

Table S4. Demographic and clinical characteristics of groups based on the sebum content.

Parameter	Sebum_High (n=106) Median	(IQR)	Sebum_Low (n=106) Median	(IQR)	*p*
Age (years)	42.50	(35.00–50.00)	59.00	(50.00–63.00)	***
BMI	20.62	(19.11–23.21)	20.81	(18.86–23.43)	
pH	6.29	(5.72–6.86)	6.30	(5.91–6.75)	
Sebum content (μg/cm^2)	22.00	(17.25–31.00)	2.00	(1.00–3.00)	***
TEWL (g/h/m^2)	11.60	(8.83–17.07)	10.95	(9.10–13.47)	
Capacitance (a.u.)	37.30	(25.00–46.15)	33.55	(25.23–40.15)	
Conductance (μS)	224.00	(182.50–291.75)	205.50	(146.25–257.50)	*
Color					
L*	58.60	(57.10–60.10)	58.15	(56.55–60.58)	
a*	5.89	(4.99–6.82)	5.25	(4.33–6.46)	*
b*	17.30	(15.93–18.88)	17.40	(15.60–18.58)	
Texture					
Number (N/mm)	0.72	(0.41–1.10)	0.67	(0.37–1.10)	
Volume ratio (μm^3/mm^2/100)	24.35	(13.95–34.40)	24.05	(13.65–34.02)	
Depth (μm)	57.10	(55.23–59.40)	57.20	(55.32–59.68)	
Surface morphology (N/cm^2)					
Color unevenness	22.48	(15.38–34.88)	19.04	(10.32–32.54)	
Spots	0.92	(0.62–1.27)	1.00	(0.64–1.45)	
Porphyrins	56.94	(33.61–75.37)	7.45	(4.32–17.23)	***
UV Spots	5.90	(5.02–6.43)	5.87	(5.21–6.40)	
Red areas	0.06	(0.03–0.12)	0.06	(0.03–0.11)	
Brown spots	1.02	(0.76–1.59)	1.85	(1.24–2.71)	***
Pores	29.54	(22.70–40.73)	17.95	(12.13–25.12)	***
Total copy number (N/cm^2)	330957.13	(170904.91–777654.96)	114291.22	(57759.63–216700.98)	***
α-diversity					
Evenness	0.32	(0.22–0.43)	0.61	(0.46–0.70)	***
Faith pd	28.87	(22.12–32.79)	45.85	(38.66–53.59)	***
Observed ASVs	190.50	(123.25–256.00)	401.50	(286.75–486.00)	***
Shannon index	2.44	(1.60–3.35)	5.30	(3.76–6.20)	***

Subgrouping was based on the sebum content (ranked and evenly divided by the number of participants). Values are presented as the median and IQR. The total copy number was measured by qPCR, as 16S rDNA gene copy numbers per cm^2. Statistical analyses between subgroups were performed using the Mann–Whitney *U* test.

Abbreviations: BMI, body mass index; TEWL, transepidermal water loss; L*, a*, b*, colorimetric parameters (lightness, red–green, and yellow–blue, respectively); UV, ultraviolet; pd, phylogenetic diversity; ASVs, amplicon sequence variants. * $p < 0.05$, *** $p < 0.001$.

Figure S2. Relationship between TEWL and observed ASVs.

(a-b) Correlation plot between TEWL and observed ASVs in each subgroup based on sebum content **(a)** and menopausal status **(b)**. The red line indicates the regression line. **(c)** Box plots showing the distribution and median of TEWL. Statistical analyses between subgroups were performed using the Kruskal–Wallis test, followed by the Steel–Dwass post-hoc test.

Abbreviations: TEWL, transepidermal water loss; ASVs, amplicon sequence variants; r, Spearman's rank correlation coefficient.

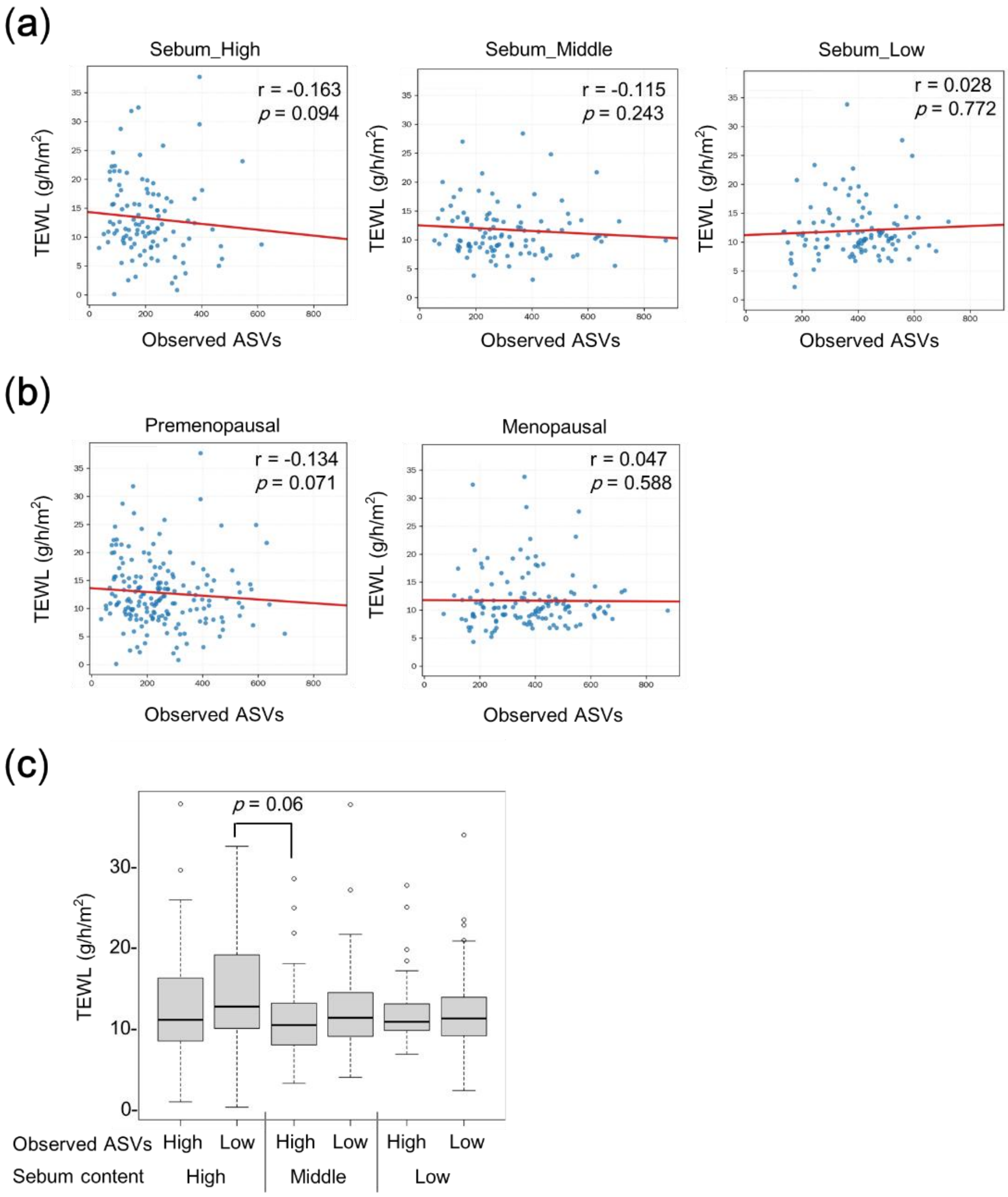

Supplement: Supplementary file 1 [file 41598_2026_59379_MOESM1_ESM.pdf]
